# Novel genetic variants in differentiated thyroid cancer and assessment of the cumulative risk

**DOI:** 10.1038/srep08922

**Published:** 2015-03-10

**Authors:** Gisella Figlioli, Bowang Chen, Rossella Elisei, Cristina Romei, Chiara Campo, Monica Cipollini, Alfonso Cristaudo, Franco Bambi, Elisa Paolicchi, Per Hoffmann, Stefan Herms, Michał Kalemba, Dorota Kula, Susana Pastor, Ricard Marcos, Antonia Velázquez, Barbara Jarząb, Stefano Landi, Kari Hemminki, Federica Gemignani, Asta Försti

**Affiliations:** 1 Molecular Genetic Epidemiology, German Cancer Research Center (DKFZ), Heidelberg, Germany; 2 Department of Biology, University of Pisa, Pisa, Italy; 3 Department of Endocrinology and Metabolism, University of Pisa, Pisa, Italy; 4 Blood Centre, Azienda Ospedaliero Universitaria A. Meyer, Firenze, Italy; 5 Department of Genomics, Life and Brain Center, University of Bonn, Bonn, Germany; 6 Institute of Human Genetics, University of Bonn, Bonn, Germany; 7 Division of Medical Genetics, University Hospital Basel; Department of Biomedicine, University of Basel, Basel, Switzerland; 8 Department of Nuclear Medicine and Endocrine Oncology, Maria Sklodowska-Curie Memorial Cancer Center and Institute of Oncology, Gliwice Branch, Gliwice, Poland; 9 Grup de Mutagènesi, Departament de Genètica i de Microbiologia, Facultat de Biociències, Universitat Autònoma de Barcelona, Cerdanyola del Vallés, Barcelona, Spain; 10 CIBER Epidemiologia y Salud Pública, ISCIII, Madrid, Spain; 11 Center for Primary Health Care Research, Clinical Research Center, Lund University, Malmö, Sweden

## Abstract

A genome-wide association study (GWAS) performed on a high-incidence Italian population followed by replications on low-incidence cohorts suggested a strong association of differentiated thyroid cancer (DTC) with single nucleotide polymorphisms (SNPs) at 9q22.33, 2q35, 20q11.22-q12 and 14q24.3. Moreover, six additional susceptibility *loci* were associated with the disease only among Italians. The present study had two aims, first to identify *loci* involved in DTC risk and then to assess the cumulative effect of the SNPs identified so far in the Italian population. The combined analysis of the previous GWAS and the present Italian study provided evidence of association with rs7935113 (*GALNTL4*, OR = 1.36, 95%CI 1.20–1.53, *p-value* = 7.41 × 10^−7^) and rs1203952 (*FOXA2*, OR = 1.29, 95%CI 1.16–1.44, *p-value* = 4.42 × 10^−6^). Experimental ENCODE and eQTL data suggested that both SNPs may influence the closest genes expression through a differential recruitment of transcription factors. The assessment of the cumulative risk of eleven SNPs showed that DTC risk increases with an increasing number of risk alleles (*p-trend* = 3.13 × 10^−47^). Nonetheless, only a small fraction (about 4% on the disease liability scale) of DTC is explained by these SNPs. These data are consistent with a polygenic model of DTC predisposition and highlight the importance of association studies in the discovery of the disease hereditability.

Thyroid cancer (TC) is an endocrine tumor arising from the parafollicular cells (medullary thyroid cancer, MTC) or the follicular cells (differentiated thyroid cancer, DTC) of the thyroid gland. Although it is relatively rare, it is the most common endocrine tumor, showing a relatively high incidence in Italy where an age standardized rate (ASR) of 13.5/100,000 was reported (http://eco.iarc.fr/eucan).

The best ascertained risk factor for DTC initiation and progression is exposure to ionizing radiation^1^. Family history and inherited genetic variants play also an important role in the disease, as demonstrated by family linkage studies, candidate-gene association studies, and genome-wide association studies (GWASs)^2^.

Risk *loci* at 2q35 (*DIRC3*), 9q22.33 (*FOXE1*) and 14q13.3 (*NKX2-1*) were identified in GWASs carried out on an Icelandic population and confirmed across different populations^3^,^4^,^5^,^6^,^7^,^8^,^9^,^10^. In 2013, we reported the results of a GWAS based on a high-incidence Italian population and the previous associations for 9q22.33 and 2q35 *loci* were confirmed in the Italian cohort and in the combined analysis of four different cohorts (Italian, Polish, Spanish and UK), respectively. Moreover, in the first replication study risk *loci* at 3q25.32 (*RARRES1*), 7q21 (*IMMP2L*), and 9q34.3 (*SNAPC4/CARD9*) were associated with DTC only among Italians. In the second study based on the Italian GWAS, a strong relationship of DTC predisposition was found with SNPs on 20q11.22-q12 (*DHX35*) and 14q24.3 (*BATF*) across different populations. In addition, 5q14 (*ARSB*) and 13q12.12 (*SPATA13*) were associated with the disease only among Italians. These results supported the idea of genetic heterogeneity between populations and suggested the hypothesis of Italian-specific DTC susceptibility alleles^11^,^12^.

In this report, 32 SNPs selected from the previous Italian GWAS were analyzed in a large cohort and the functional role of the best associated SNPs was investigated by using ENCODE project data and by expression quantitative trait *loci* (eQTL) analyses. Furthermore, results from the present analysis were combined with those obtained in the previous Italian studies and the cumulative effect of the GWAS-identified SNPs on DTC risk was evaluated.

## Results

In the first phase of this study 32 SNPs were replicated in a large Italian cohort consisting of 1,539 DTC cases and 1,719 controls (the study populations are described in Kohler et al. (2013)^11^ and in Supplementary Table S1). Results obtained in this phase and in the previous GWAS are reported in Supplementary Table S2. One marker (rs1358175) demonstrated deviation from Hardy-Weinberg Equilibrium in controls (*p-value* < 0.005) and was excluded from the analysis. A statistically significant association at *p-value* < 0.05 and at the same direction as in the GWAS was found for rs10864251, rs4908581, rs1400967, rs290219, rs7935113, rs4624074 and rs1203952. Additionally, an association with DTC in the same direction of the GWAS was obtained for rs11130536 and rs3863973, although not significant.

Combining the GWAS data with the present Italian results (2,260 cases and 2,218 controls), SNPs rs7935113 (OR = 1.36, 95% CI 1.20–1.53, *p-value* = 7.41 × 10^−7^) and rs1203952 (OR = 1.29, 95% CI 1.16–1.44, *p-value* = 4.42 × 10^−6^) reached close to a genome-wide significance (Table 1). However, these strong associations were not confirmed in the replication studies on the Polish and the Spanish populations. Consequently, the combined analysis of all replication studies (Italian, Polish, Spanish) and the joint analysis of all cohorts (GWAS, Italian, Polish, Spanish) did not reach a genome-wide significance, in agreement with a high heterogeneity between the study populations. None of the remaining *loci* were associated with the disease (Table 1).

**Figure 1:**
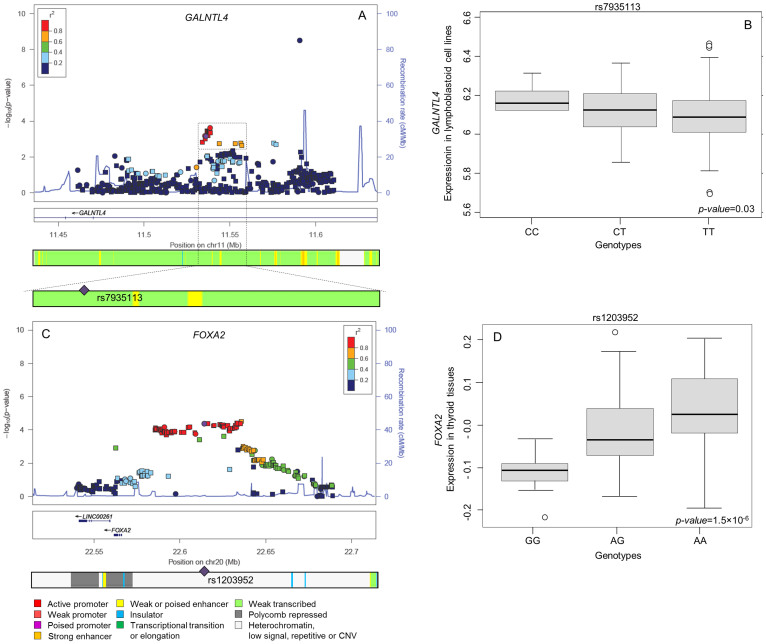
Regional association plots and functional prediction of the strongest associated variants. On the left (A and C) the regional plots are shown. In each plot, -log(10)p-value (y-axis) of SNPs are shown according to their chromosomal position (x-axis). SNP of interest is indicated by a violet circle. SNPs that were genotyped in the GWAS are marked by circles; imputed SNPs are marked as squares. The color of the SNPs represents the strength of the linkage disequilibrium with the SNP of interest. Blue line indicates local recombination rate (cM/Mb). On the bottom, the chromatin state segmentation profile (ChromHMM) in lymphoblastoid cell-line is reported. On the right (B and D), the correlation between gene expression levels and SNP genotypes are shown according to the data available at SNPexp (http://tinyurl.com/snpexp) on lymphoblastoid cell lines (B) and at the GTEx Portal (http://www.gtexportal.org/home/) on thyroid tissues (D).

To increase the knowledge about the associated regions imputation over 200 Kb intervals spanning the associated *loci* were performed. At 11p15.3, in the intronic region of the *GALNTL4* gene, the LD block including the index SNP rs7935113 defined the association. This block is located in a weak transcribed region in GM12878 cell line (Figure 1A). HaploReg v2 analysis revealed that rs7935113 risk allele removes a MZF1 transcription factor binding site (TFBS) and creates a SRF TFBS. This variation also affects the chromatin structure and enhancer histone markers on different cell lines as demonstrated by the ENCODE project data (Supplementary Table S3). eQTL analysis on lymphoblastoid cells demonstrated a significant association between rs7935113 and *GALNTL4* expression level (Figure 1B; *p-value* = 0.03). However, this correlation was not found in thyroid tissues (data not shown). Several functional roles were also predicted for SNPs in LD with rs7935113 due to location in enhancer histone marker sites in different cell lines (e.g. GM12878, HSMM and HMEC) and alterations of TFBSs (Supplementary Table S3).

At 20p11, a large region near *FOXA2* is associated with low *p-values* to DTC risk, as indicated by the index SNP rs1203952, by GWAS typed SNPs (e.g. rs910956, rs2424440 and rs1203930) and by imputed SNPs (e.g. rs4815130, rs747912, rs910957 and rs1203953). The ChromHMM on GM12878 cells did not show any functional role for this region (Figure 1C). However, rs1203952, located 52 kb upstream of *FOXA2*, is predicted to alter the binding of Evi-1, Foxp1, Pou2f2 and SIX5 TFs and of the regulatory protein FOXA1 and to have an effect on the chromatin structure in MCF-7 cells. Similar roles were predicted for SNPs in LD with the index SNP rs1203952. Interestingly, according to the GTex data, SNPs belonging to this LD block are associated with a differential expression of *FOXA2* gene in thyroid tissues (*p-value* = 1.5 × 10^−6^) (Figure 1D and Supplementary Table S3).

**Figure 2:**
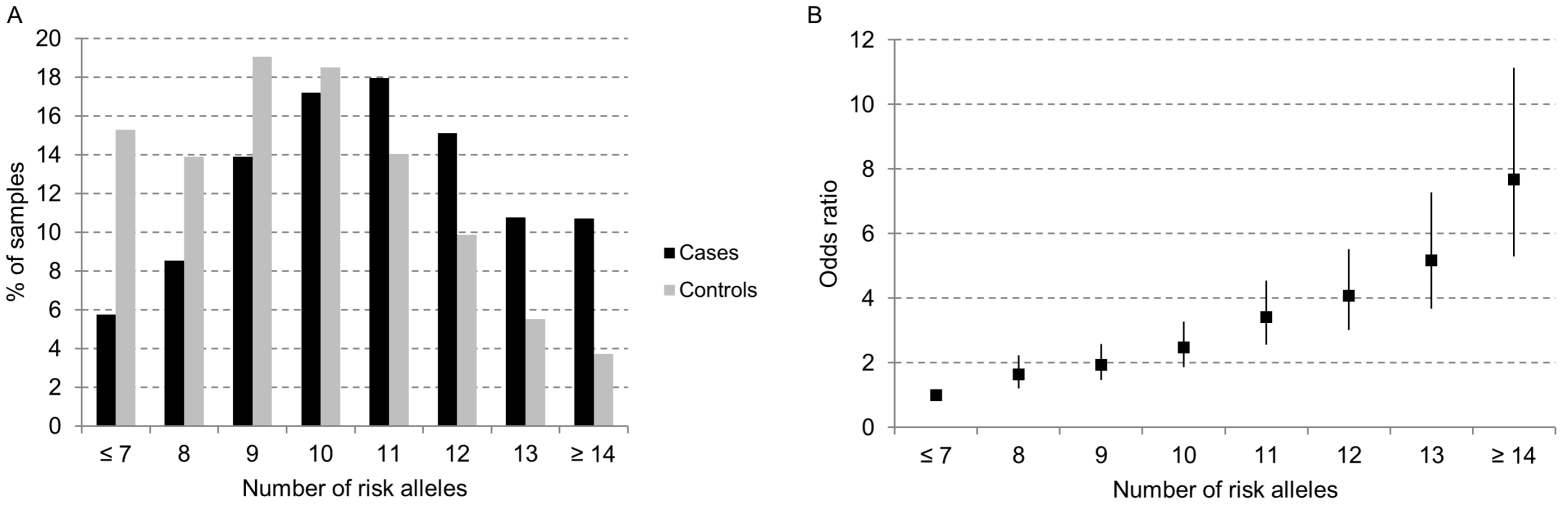
Cumulative risk assessment. (A) Sample distribution according to the number of risk alleles in eleven SNPs associated in the Italian DTC cases (black columns) and controls (grey columns). (B) Plot of the increasing ORs for DTC with increasing number of risk alleles. The category ≤7 was chosen as reference (OR = 1.0); vertical bars correspond to 95% confidence intervals.

We investigated the cumulative effect of 11 independent susceptibility SNPs in 10 genes (*DIRC3*, *IMMP2L*, *RARRES1*, *SNAPC4/CARD9*, *BATF*, *DHX35*, *ARSB*, *SPATA13*, *GALNTL4*, *FOXA2*) in the Italian population (1,791 cases and 1,588 controls; Supplementary Table S4). The risk allele distribution in both cases and controls followed a normal distribution, but with a shift toward a higher number of risk alleles among cases (Figure 2A). A dose-dependent increase in risk of DTC was observed with an increasing number of risk alleles (OR per allele = 1.30, 95% CI 1.26–1.35; *p-trend* = 3.13 × 10^−47^). Compared to those with ≤7 risk alleles individuals with ≥14 risk alleles had 7.68 times higher risk of getting DTC (Supplementary Table S5, Figure 2B). As the well-established *FOXE1* SNP rs965513 was genotyped only in the GWAS samples, we analyzed the effect of all the 12 SNPs associated with DTC in the Italian population in these samples (642 cases and 416 controls). The same trend was observed (OR per allele = 1.52, 95% CI 1.42–1.63; *p-trend* = 1.71 × 10^−32^) with individuals carrying ≥14 risk alleles having a 27.45 times higher risk of DTC than those with ≤7 risk alleles (Supplementary Table S6 and Supplementary Figure S1). This 3.5 times higher risk after including the *FOXE1* SNP is explained by the concept of a “winners curse”, which implies that by chance the first reported association is higher than the one obtained in the replication. Finally, we estimated the proportion of variance in DTC risk (on the liability scale) explained by the identified susceptibility SNPs. We assumed a prevalence of DTC among Italians to be 0.01 and found that the 12 SNPs explained about 4% of the disease risk. The *FOXE1* SNP alone explained less than 1% of the risk.

## Discussion

The present study had two main goals: to identify novel SNPs predisposing to DTC and to assess the cumulative effect of the SNPs identified so far in the Italian population. We analyzed 32 SNPs from the *loci* that showed evidence of association with DTC in our recent GWAS. The associations between DTC and SNPs rs7935113 and rs1203952 reached close to a genome-wide significance in the combined Italian populations. Risk of the disease widely increased with increasing number of risk alleles, which altogether explained about 4% of the genetic variance in DTC susceptibility.

The common variant rs7935113 is located in an intronic region of *GALNTL4* at 11p15.3. This gene is a member of a large subfamily of the galactosaminyltransferases (GalNAc-Ts). Although little is known about its biological function, it is well established that abnormal O-glycan production contributes to the malignant phenotype and plays an important role in cell adhesion, invasion, and metastasis^13^. Our eQTL analysis indicated that the rs7935113-C risk allele leads to an increased expression of *GALNTL4*. This is consistent with previous data which found various genes encoding GALNAC-Ts to be overexpressed in malignant tissues compared with normal tissue, such as *GALNT3* in pancreatic cancer and *GALNT6* in breast cancer^14^,^15^. Moreover, rs7935113 alters the binding sites of SRF and MZF1 transcription factors. To date, various studies have shown that SRF is involved in important cellular processes such as expression of tissue-specific genes, cell proliferation, differentiation, and apoptosis^16^,^17^,^18^,^19^. SRF has been found significantly up-regulated in PTC and anaplastic carcinoma as compared to non-tumor thyroid tissues. Moreover, *in vitro* assays have indicated that overexpression of SRF in thyroid cancer cells enhances the expression level of c-Fos protein, cell migration, and invasiveness^20^. The role of MZF1 in thyroid cancer has never been investigated, however, it was involved in colorectal, cervical, and bladder carcinogenesis^21^,^22^,^23^.

rs1203952 is located on 20p11, upstream of *FOXA2* (also known as *HNF3B*), belonging to the same forkhead-box (FOX) TF family of *FOXE1*, the strongest risk gene associated with DTC so far. As *FOXE1*, *FOXA2* is able to bind *TPO* promoter and to modulate its transcriptional activity^24^. Thus, *FOXE1* and *FOXA2* could act together in the regulation of thyroid hormones triiodothyronine (T_3_) and thyroxine (T_4_) synthesis. The eQTL analysis demonstrated that homozygocity for rs1203952-G risk allele is associated with a decreased level of *FOXA2* expression in thyroid tissues. This result is consistent with a previously published study, showing that thyroid cancer cells had a decreased expression of *FOXA2* when compared to normal cells, and *FOXA2* forced re-expression was associated with cell growth inhibition^25^. Interestingly, *FOXA2* is a methylated gene in breast and lung cancer cells and its overexpression in a lung cancer cell line led to growth arrest and apoptosis^26^,^27^. The down-regulation of *FOXA2* expression in thyroid tissues could be in part explained by the action of the regulatory protein FOXA1 and of the TFs Evi-1, Foxp1, Pou2f2 and SIX5, which binding sites are altered by rs1203952 and for which a role in cell proliferation and differentiation has already been reported^28^,^29^,^30^,^31^,^32^. In particular, the mechanism of action of FOXA1 was described also in anaplastic TC, where it regulates the expression of the cell cycle inhibitor p27^kip1^ and promotes cell proliferation^33^.

Given these observations, rs7935113 and rs1203952 could lead to DTC development and progression by altering *GALNTL4* and *FOXA2* expression through a different recruitment of TFs. Besides these mechanisms, many genetic variants in the LD block containing rs7935113 and rs1203952 could alter the binding of proteins and the function of regulatory elements (e.g. enhancers), suggesting that many other transcriptional regulatory mechanisms could explain the identified association signals. Fine-mapping studies of 11p15.3 and 20p11 *loci* are warranted to check whether other functional variants, including rare genetic variants, could explain the observed associations.

The second objective of this work was to determine the combined effect of the risk variants identified so far in the Italian population on DTC risk. We showed that, although individual susceptibility alleles have only a modest effect on the disease, the risk widely increases when the alleles are combined. The additive effect of the SNP alleles on DTC risk was also recently reported for five GWAS-identified SNPs (rs965513, rs944289, rs966423, rs2439302, and rs116909374) in Polish and Ohio cohorts^6^. Moreover, our results suggested that the variation in DTC risk in the Italian population is explained in part by the 12 SNPs identified so far (about 4%). We note that this value most likely represents the lower bound for the contribution of these 12 loci, although this type of approaches that calculate heritability of disease liability have been recommended for the estimation of the additive genetic contribution of common SNPs in a complex disease susceptibility^34^. The low proportion of genetic variance explained by the identified risk alleles and the small proportion of a general population that is expected to be in the highest risk group, less than 5% in the Italian control population had ≥14 risk alleles, restrict currently the usefulness of genetic data in the clinical practice. Taken together these data are consistent with a multifactorial and polygenic model of DTC susceptibility. Future genetic research on larger sample sets and novel technologies, such as array-based fine-mapping and next generation sequencing, are warranted to identify rare high-penetrance variants and gene-gene interactions that could explain a higher percentage of genetic and phenotypic variance in DTC.

In conclusion our study provides further insights into inherited susceptibility to DTC among Italians and highlights the importance of genetic association studies.

## Methods

### Ethics statement

Study participants were recruited according to the protocols approved by the institutional review boards in accordance with the Declaration of Helsinki. All subjects provided written informed consent to participate in the study.

### Study populations

This study was conducted on three sets of samples (Italian, Polish and Spanish), which were described elsewhere^11^ and reported in Supplementary Table S1. All cases and controls were of Caucasian origin. Briefly, the Italian replication cohort included 1,539 DTC patients attending the University Hospital Cisanello in Pisa. The control group (1,719) was recruited from individuals without any thyroid disease and cancer history: 1,079 were workers of the same hospital of Pisa and 640 were blood donors from the Meyer Hospital in Florence. The Polish group comprised of 468 DTC patients and 470 healthy controls from the Department of Nuclear Medicine and Endocrine Oncology, Maria Sklodowska-Curie Memorial Cancer Center and Institute of Oncology in Gliwice. The Spanish cohort consisted of 446 DTC cases, recruited by the Department of Genetics and Microbiology of Autonomous University of Barcelona, and 420 healthy individuals.

DNA isolated from peripheral blood leukocytes (Italian, Polish and Spanish cohorts) and oral mucosa cells (Spanish cohort) were used. DNA was extracted according to the protocols used in respective institutions which provided the samples. DNA concentration was evaluated with NanoDrop spectrophotometer. For Italian and Polish samples, whole genome amplification was performed using Illustra GenomiPhi V2 DNA Amplification Kit (GE Healthcare) according to the manufacturer's protocol.

### SNP selection and genotyping

Candidate SNPs were selected based on the results of the Italian GWAS reported by Köhler and coworkers where the best 250 SNPs were already investigated^11^,^12^. Here, the following 250 SNPs were visually screened for the quality of their clustering pattern. The Manhattan plots (±100 kb from the SNP position) were also investigated and the SNPs were screened for other SNPs in linkage disequilibrium (LD) with the variant of interest. Finally, we selected 32 SNPs for further evaluation. All of them represented a region with at least two SNPs associated with DTC.

Genotyping was carried out using the TaqMan SNP genotyping assays (Life Technologies) according to the manufacturer's guidelines. To assure the genotyping reliability, repeated analysis was performed in a randomly selected 10% of samples (the average concordance rate was 99%). After excluding samples with more than 50% missing genotypes, all markers had a call rate greater than 95%, with a mean call rate of 98%. Original GWAS samples were re-genotyped (rs7935113 and rs1203952) and the results confirmed the GWAS data (concordance > 99%).

### Statistical analysis

We tested the genotype distributions in controls for Hardy-Weinberg equilibrium by using the chi-square test. For each SNP logistic regression analysis was performed to determine allelic odds ratios (ORs) with 95% confidence intervals (95% CIs) and allelic *p-values*. The calculations were done for each cohort separately for unadjusted models as well as with adjustment for sex and age/age at diagnosis. The results for adjusted models were similar to the unadjusted ones and are not reported. For Italian cohorts, further adjustments for the enrollment center (University Hospital Cisanello in Pisa and Meyer Hospital in Florence) or the place of birth (Southern Italy or Northern and Central Italy) did not substantially change the associations. For all replication studies combined, calculations were carried out correcting for age, sex and cohort. The Cochran's Q-statistics was calculated to test for heterogeneity and the *I*^*2*^ statistics to quantify the proportion of the total variation due to heterogeneity. All these analyses were performed using SAS version 9.2 (SAS Institute In., Cary, NC, USA).

### Computational analysis

To evaluate the associated *loci* more thoroughly, we imputed genotypes of all SNPs that were not genotyped in the GWAS located 100 Kb upstream or downstream of the most significant SNP. We employed genotype information from the CEU panels of the publicly available HapMap3 (www.hapmap.org/) and 1000 Genomes Project (www.1000genomes.org/) databases. We used the software IMPUTE2 to perform imputation analysis on each associated *locus*. Regional plots were generated using LocusZoom (http://csg.sph.umich.edu/locuszoom/).

We searched SNPs in high LD (r^2^ ≥ 0.8) with SNPs that showed the strongest associations with DTC predisposition based on the CEU data of the 1000 Genomes Project pilot release (www.1000genomes.org/). To explore the epigenetic profile of the best associated regions, we checked the chromatin state segmentation profile (ChromHMM) in lymphoblastoid cells (GM12878) generated by the ENCODE project and available at the UCSC Genome Browser (http://genome.ucsc.edu/). To assess the possible functional role of each SNP we used the ENCODE-based tool HaploReg v2 (www.broadinstitute.org/mammals/haploreg). In addition, we examined the eQTL data available for lymphoblastoid cells and thyroid tissues by using SNPexp (http://tinyurl.com/snpexp) and GTEx Portal (http://www.gtexportal.org/home/), respectively.

### Assessment of cumulative risk

We assessed the cumulative effect of the independent significant SNPs identified in our previous analyses and in the present study in the Italian population. For each SNP the genotypes were coded as 0, 1 or 2 indicating the number of risk alleles in the genotype and individuals were grouped into categories based on the number of risk alleles (≤7, 8, 9, 10, 11, 12, 13 and ≥14). To avoid any bias due to missing data, samples with one or more missing genotypes were not included. ORs were calculated comparing the groups defined by varying number of risk alleles to the group with the lowest number of risk alleles. This analysis was performed by Statgraphics Centurion software (StatPoint, USA) and the R program. Additionally, we used the Genome-wide Complex Trait Analysis (GCTA) program (http://www.complextraitgenomics.com/software/gcta/) to calculate the proportion of DTC variance (using a liability model) that is explained by the significant SNPs identified so far in the Italian population^35^. In a sample of independent individuals, this method uses a random effects mixed linear model to compare a matrix of pairwise genomic similarity with a matrix of pairwise phenotypic similarity.

## Author Contributions

A.F., F.G., K.H., S.L., B.J. and A.V. organized and designed the study. G.F., C.C., P.H. and S.H. performed the experiments. G.F., B.C., A.F. and S.L. contributed to the design and execution of statistical analysis. R.E., C.R., M.C., A.C., B.J., M.K., D.K., A.V., S.P. and R.M. contributed to the collection of samples. F.B., E.P., M.K., D.K., S.P. and R.M. provided the administrative, technical, and material support. G.F., A.F., F.G. and K.H. wrote and reviewed the manuscript. A.F., F.G., K.H., S.L. and RE supervised the study.

## Supplementary Material

Supplementary Information Supplementary Information

## Figures and Tables

**Table 1:** Risk of differentiated thyroid cancer in all cohorts

SNP	Chr	Position	Gene	Risk allele	Population	Number of cases/controls	Risk allele frequency (case/controls)	Allelic OR (95% CI)^(^*^)^	*p-value*^(^*^)^
rs10864251	1	6934392	*CAMTA1*	C	GWAS	648/431	0.45/0.37	1.40 (1.17–1.66)	2.06 × 10^−4^
					Italian	1482/1529	0.45/0.41	1.17 (1.06–1.29)	2.87 × 10^−3^
					Polish	452/431	0.37/0.41	0.85 (0.70–1.02)	0.08
					Spanish	354/404	0.36/0.40	0.85 (0.69–1.05)	0.13
					Italian cohorts	2130/1960	-	1.17 (1.06–1.29)	1.40 × 10^−3^
					All replications	2288/2364	-	0.99 (0.91–1.08)	0.87
					JOINT	2936/2795	-	1.05 (0.97–1.14)	0.19
								*P_het_* = 2 × 10^−4^, *I*^*2*^ = 84.37	
rs4908581	1	6940572	*CAMTA1*	A	GWAS	649/431	0.60/0.52	1.41 (1.19–1.68)	9.35 × 10^−5^
					Italian	1367/1458	0.60/0.56	1.16 (1.04–1.29)	5.93 × 10^−3^
					Polish	448/432	0.57/0.60	0.87 (0.72–1.05)	0.14
					Spanish	345/398	0.50/0.55	0.84 (0.69–1.03)	0.09
					Italian cohorts	2016/1889	-	1.22 (1.11–1.35)	4.61 × 10^−5^
					All replications	2160/2288	-	1.01 (0.92–1.10)	0.88
					JOINT	2809/2719	-	1.09 (1.00–1.18)	0.04
								*P_het_* = 1 × 10^−4^, *I*^*2*^ = 86.19	
rs1400967	2	123205807	*LOC728241*	T	GWAS	649/430	0.27/0.19	1.52 (1.23–1.87)	8.43 × 10^−5^
					Italian	1447/1554	0.23/0.20	1.17 (1.03–1.32)	0.01
					Polish	454/437	0.19/0.20	0.97 (0.77–1.23)	0.83
					Spanish	364/408	0.22/0.25	0.86 (0.67–1.08)	0.19
					Italian cohorts	2096/1984	-	1.22 (1.09–1.36)	7.11 × 10^−4^
					All replications	2265/2399	-	1.01 (0.91–1.13)	0.81
					JOINT	2914/2829	-	1.11 (1.01–1.22)	0.03
								*P_het_* = 2.1 × 10^−3^, *I*^*2*^ = 79.53	
rs11130536	3	56643562	*C3orf63*	C	GWAS	648/431	0.84/0.77	1.51 (1.22-1.88)	1.73 × 10^−4^
					Italian	1488/1569	0.82/0.80	1.14 (1.00-1.29)	0.05
					Polish	456/444	0.75/0.73	1.10 (0.90-1.36)	0.35
					Spanish	352/409	0.76/0.75	1.03 (0.81-1.30)	0.82
					Italian cohorts	1068/2000	-	1.24 (1.10-1.40)	3.27 × 10^−4^
					All replications	2296/2422	-	1.10 (0.99-1.23)	0.07
					JOINT	2944/2853	-	1.17 (1.06-1.28)	1.38 × 10^-3^
								*P_het_* = 0.07, *I*^*2*^ = 57.67	
rs3863973	3	73581688	*PDZRN3*	G	GWAS	649/431	0.26/0.19	1.49 (1.20–1.84)	2.14 × 10^−4^
					Italian	1457/1559	0.22/0.20	1.13 (1.00–1.28)	0.06
					Polish	448/431	0.22/0.19	1.25 (0.99–1.58)	0.06
					Spanish	315/394	0.21/0.21	0.99 (0.76–1.27)	0.91
					Italian cohorts	2106/1990	-	1.22 (1.09–1.37)	5.95 × 10^−4^
					All replications	2220/2384	-	1.12 (1.00–1.24)	0.05
					JOINT	2869/2815	-	1.20 (1.09–1.32)	1.75 × 10^−4^
								*P_het_* = 0.07, *I*^*2*^ = 58.18	
rs290212	9	92635888	*SYK*	C	GWAS	649/431	0.33/0.26	1.44 (1.19–1.75)	1.86 × 10^−4^
					Italian	1476/1575	0.30/0.27	1.13 (1.01–1.26)	0.03
					Polish	455/423	0.22/0.25	0.88 (0.71–1.10)	0.26
					Spanish	342/406	0.24/0.25	0.93 (0.74–1.18)	0.56
					Italian cohorts	2125/2006	-	1.23 (1.11–1.37)	6.84 × 10^−5^
					All replications	2273/2404	-	1.05 (0.95–1.16)	0.31
					JOINT	2922/2835	-	1.13 (1.04–1.24)	5.10 × 10^−3^
								*P_het_* = 3.7 × 10^−3^, *I*^*2*^ = 77.71	
rs7935113	11	11492456	*GALNTL4*	C	GWAS	647/431	0.23/0.16	1.50 (1.20–1.88)	3.26 × 10^−4^
					Italian	1454/1572	0.20/0.16	1.28 (1.12–1.46)	2.20 × 10^−4^
					Polish	452/443	0.13/0.13	0.99 (0.75–1.30)	0.93
					Spanish	352/407	0.18/0.18	1.02 (0.78–1.32)	0.90
					Italian cohorts	2101/2003	-	1.36 (1.20–1.53)	7.41 × 10^−7^
					All replications	2258/2422	-	1.18 (1.05–1.33)	4.77 × 10^−3^
					JOINT	2905/2853	-	1.24 (1.12–1.38)	2.71 × 10^−5^
								*P_het_* = 0.05, *I*^*2*^ = 61.96	
rs4624074	14	33814177	*C14orf147*	T	GWAS	649/431	0.58/0.49	1.41 (1.19–1.68)	8.62 × 10^−5^
					Italian	1468/1560	0.56/0.53	1.12 (1.01–1.24)	0.03
					Polish	436/423	0.57/0.56	1.05 (0.86–1.27)	0.65
					Spanish	301/405	0.54/0.55	0.97 (0.79–1.20)	0.80
					Italian cohorts	2117/1991	-	1.20 (1.09–1.32)	1.46 × 10^−4^
					All replications	2205/2388	-	1.06 (0.97–1.16)	0.19
					JOINT	2854/2819	-	1.13 (1.05–1.22)	2.25 × 10^−3^
rs1203952	20	22562132	*FOXA2*	G	GWAS	647/429	0.28/0.21	1.49 (1.21–1.83)	1.37 × 10^−4^
					Italian	1453/1514	0.27/0.23	1.25 (1.11–1.41)	2.10 × 10^−4^
					Polish	453/437	0.21/0.23	0.88 (0.70–1.10)	0.25
					Spanish	351/404	0.23/0.21	1.10 (0.86–1.40)	0.44
					Italian cohorts	2100/1943	-	1.29 (1.16–1.44)	4.42 × 10^−6^
					All replications	2257/2355	-	1.13 (1.02–1.25)	0.02
					JOINT	2904/2784	-	1.20 (1.09–1.31)	1.20 × 10^−4^
								*P_het_* = 6.1 × 10^−3^, *I*^*2*^ = 75.80	

(*)For each analyzed cohort unadjusted allelic odds ratios (ORs) with corresponding 95% confidence intervals (CIs) and *p-values* are shown; the joint analysis of two or more cohorts was adjusted for age, sex and cohort.
