# Supplementary material for: Novel genetic variants in differentiated thyroid cancer and assessment of the cumulative risk
Source: Sci Rep. 2015 Mar 10;5:8922. doi: 10.1038/srep08922 (PMC4354074; doi:10.1038/srep08922)
Supplement: Supplementary Information [file srep08922-s1.pdf]

Novel genetic variants in differentiated thyroid cancer and assessment of the cumulative risk

Gisella Figlioli[1,2], Bowang Chen[1], Rossella Elisei[3], Cristina Romei[3], Chiara Campo[1,2], Monica Cipollini[2], Alfonso Cristaudo[3], Franco Bambi[4], Elisa Paolicchi[2], Per Hoffmann[5,6,7], Stefan Herms[5,6,7], Michał Kalemba[8], Dorota Kula[8], Susana Pastor[9,10], Ricard Marcos[9,10], Antonia Velázquez[9,10], Barbara Jarząb[8], Stefano Landi[2*], Kari Hemminki[1,11], Federica Gemignani[2†], Asta Försti[1,11*†]

[1]Molecular Genetic Epidemiology, German Cancer Research Center (DKFZ), Heidelberg, Germany. [2]Department of Biology, University of Pisa, Pisa, Italy. [3]Department of Endocrinology and Metabolism, University of Pisa, Pisa, Italy. [4]Blood Centre, Azienda Ospedaliero Universitaria A. Meyer, Firenze, Italy. [5]Department of Genomics, Life and Brain Center, University of Bonn, Bonn, Germany. [6]Institute of Human Genetics, University of Bonn, Bonn, Germany. [7]Division of Medical Genetics, University Hospital Basel; Department of Biomedicine, University of Basel, Basel, Switzerland. [8]Department of Nuclear Medicine and Endocrine Oncology, Maria Sklodowska-Curie Memorial Cancer Center and Institute of Oncology, Gliwice Branch, Gliwice, Poland. [9]Grup de Mutagènesi, Departament de Genètica i de Microbiologia, Facultat de Biociències, Universitat Autònoma de Barcelona, Cerdanyola del Vallés, Barcelona, Spain. [10]CIBER Epidemiologia y Salud Pública, ISCIII, Madrid, Spain. [11]Center for Primary Health Care Research, Clinical Research Center, Lund University, Malmö, Sweden.

[†]these authors contributed equally to the manuscript

[*]**Address all correspondence and requests for reprints to:** Asta Försti, Division of Molecular Genetic Epidemiology, German Cancer Research Center (DKFZ), Im Neuenheimer Feld 580, 69120 Heidelberg, Germany, Tel: +49 6221421803, Fax: +49 6221421810, e-mail: a.foersti@dkfz-heidelberg.de and Stefano Landi, Genetics Department of Biology, University of Pisa, Via Derna 1, 56126 Pisa, Italy, Tel: +39 0502211528, Fax: +39 0502211527, e-mail: stefano.landi@unipi.it.

Supplementary Table S1. Characteristics of all cohorts included in the study

Cohort		Number	Age (Average±SD)	Sex (M:F)[a]	Histology[b]
GWAS	Cases	701	46.10±11.32	155:539	PTC: 701
	Controls	499	51.74±10.42	115:383	-
Italian	Cases	1539	51.95±13.47	423:1113	PTC: 1330; FTC: 172; PTC/FTC: 4; ATC/PDTC: 5; Unspecified DTC: 12
	Controls	1719	55.93±15.24	1134:571	-
Polish	Cases	468	53.25±15.13	62:406	PTC: 453; FTC: 15
	Controls	470	47.93±14.40	86:384	-
Spanish	Cases	446	47.05±15.33	95:350	PTC: 378; FTC: 63 Unspecified DTC: 2
	Controls	420	47.17±17.59	170:248	-

[a]M, male; F, female; [b]ATC, anaplastic thyroid cancer; DTC, differentiated thyroid cancer, FTC, follicular thyroid cancer; PDTC, poorly differentiated thyroid cancer; PTC, papillary thyroid cancer

Supplementary Table S2. Results of the GWAS analysis and the Italian replication study for all SNPs

SNP	Chr	Position	Gene	Risk allele	Population	Risk allele frequency (cases)	Risk allele frequency (controls)	Unadjusted allelic OR (95% CI)	*p-value*
rs10864251	1	6934392	*CAMTA1*	C	GWAS	0.45	0.37	1.40 (1.17-1.66)	2.06×10^{-4}
					Italian	0.45	0.41	1.17 (1.06-1.29)	2.87×10^{-3}
rs4908581	1	6940572	*CAMTA1*	A	GWAS	0.60	0.52	1.41 (1.19-1.68)	9.35×10^{-5}
					Italian	0.60	0.56	1.16 (1.04-1.29)	5.93×10^{-3}
rs1043443	1	20849967	*PINK1*	T	GWAS	0.80	0.73	1.47 (1.20-1.80)	1.74×10^{-4}
					Italian	0.75	0.79	0.80 (0.71-0.90)	2.90×10^{-4}
rs2226023	1	208933824	*KCNH1*	A	GWAS	0.67	0.59	1.41 (1.18-1.68)	1.76×10^{-4}
					Italian	0.62	0.62	1.03 (0.92-1.14)	0.65
rs1400967	2	123205807	*LOC728241*	T	GWAS	0.27	0.19	1.52 (1.23-1.87)	8.43×10^{-5}
					Italian	0.23	0.20	1.17 (1.03-1.32)	0.01
rs6740362	2	208593899	*LOC389072*	A	GWAS	0.90	0.84	1.63 (1.26-2.10)	1.85×10^{-4}
					Italian	0.88	0.88	0.99 (0.84-1.15)	0.86
rs13021389	2	228915438	*LOC646794*	T	GWAS	0.59	0.51	1.40 (1.18-1.67)	1.28×10^{-4}
					Italian	0.58	0.56	1.09 (0.99-1.21)	0.09
rs11130536	3	56643562	*C3orf63*	C	GWAS	0.84	0.77	1.51 (1.22-1.88)	1.73×10^{-4}
					Italian	0.82	0.80	1.14 (1.00-1.29)	0.05
rs3863973	3	73581688	*PDZRN3*	G	GWAS	0.26	0.19	1.49 (1.20-1.84)	2.14×10^{-4}
					Italian	0.22	0.20	1.13 (1.00-1.28)	0.06
rs11926023	3	106082020	*LOC391562*	C	GWAS	0.31	0.23	1.47 (1.21-1.79)	1.10×10^{-4}
					Italian	0.27	0.30	0.85 (0.76-0.95)	4.21×10^{-3}
rs9846474	3	151721011	*SERP1*	T	GWAS	0.25	0.18	1.51 (1.22-1.87)	1.39×10^{-4}
					Italian	0.21	0.20	1.06 (0.93-1.20)	0.41
rs709386	5	98357950	*CHD1*	T	GWAS	0.3	0.23	1.47 (1.20-1.79)	1.43×10^{-4}
					Italian	0.26	0.26	1.00 (0.89-1.12)	0.96
rs2108237	7	106003819	*LOC730026*	A	GWAS	0.81	0.74	1.49 (1.22-1.83)	1.17×10^{-4}
					Italian	0.76	0.79	0.87 (0.77-099)	0.03
rs10091141	8	64378427	*YTHDF3*	A	GWAS	0.43	0.35	1.41 (1.18-1.69)	1.38×10^{-4}
					Italian	0.36	0.4	0.86 (0.78-0.96)	5.12×10^{-3}
rs12550873	9	92613418	*SYK*	T	GWAS	0.20	0.14	1.60 (1.26-2.03)	9.98×10^{-5}
					Italian	0.16	0.16	1.01 (0.88-1.16)	0.91
rs290212	9	92635888	*SYK*	C	GWAS	0.33	0.26	1.44 (1.19-1.75)	1.86×10^{-4}
					Italian	0.30	0.27	1.13 (1.01-1.26)	0.03
rs1120146	9	107933671	*NULL*	C	GWAS	0.64	0.56	1.41 (1.19-1.69)	1.14×10^{-4}
					Italian	0.59	0.62	0.88 (0.79-0.97)	0.01
rs10824195	10	75943585	*ADK*	A	GWAS	0.87	0.81	1.55 (1.23-1.96)	1.83×10^{-4}
					Italian	0.84	0.86	0.86 (0.75-0.99)	0.04
rs7935113	11	11492456	*GALNTL4*	C	GWAS	0.23	0.16	1.50 (1.20-1.88)	3.26×10^{-4}
					Italian	0.20	0.16	1.28 (1.12-1.46)	2.20×10^{-4}
rs12421802	11	79471375	*LOC646112*	A	GWAS	0.68	0.6	1.39 (1.17-1.67)	2.80×10^{-4}
					Italian	0.68	0.67	1.04 (0.94-1.17)	0.44
rs10459079	12	22287524	*ST8SIA1*	C	GWAS	0.61	0.53	1.41 (1.18-1.67)	1.26×10^{-4}
					Italian	0.57	0.57	1.00 (0.91-1.11)	0.95
rs1459593	12	83464476	*SLC6A15*	A	GWAS	0.13	0.07	1.82 (1.35-2.47)	8.42×10^{-5}
					Italian	0.12	0.12	0.98 (0.84-1.14)	0.80
rs1624638	13	94529493	*ABCC4*	G	GWAS	0.72	0.65	1.39 (1.16-1.68)	4.44×10^{-4}
					Italian	0.71	0.70	1.01 (0.90-1.14)	0.82
rs1729767	13	94617943	*ABCC4*	G	GWAS	0.36	0.28	1.44 (1.19-1.73)	1.46×10^{-4}
					Italian	0.35	0.34	1.05 (0.94-1.17)	0.40
rs16957649	13	100200879	*ARF4P3*	A	GWAS	0.83	0.76	1.52 (1.23-1.88)	1.05×10^{-4}
					Italian	0.81	0.81	1.02 (0.89-1.16)	0.81
rs4624074	14	33814177	*C14orf147*	T	GWAS	0.58	0.49	1.41 (1.19-1.68)	8.62×10^{-5}
					Italian	0.56	0.53	1.12 (1.01-1.24)	0.03
rs10129961	14	33837056	*C14orf147*	A	GWAS	0.31	0.23	1.46 (1.20-1.78)	1.56×10^{-4}
					Italian	0.28	0.27	1.07 (0.95-1.19)	0.26
rs1358175(*)	17	36011315	*SMARCE1*	C	GWAS	0.48	0.40	1.40 (1.17-1.66)	1.72×10^{-4}
					Italian	0.45	0.42	1.11 (1.01-1.23)	0.04
rs10409222	19	51071487	*FOXA3*	T	GWAS	0.23	0.16	1.52 (1.21-1.90)	2.32×10^{-4}
					Italian	0.18	0.18	1.01 (0.88-1.16)	0.92
rs10408163	19	52288942	*ZC3H4*	T	GWAS	0.35	0.27	1.44 (1.19-1.74)	1.60×10^{-4}
					Italian	0.33	0.31	1.07 (0.96-1.19)	0.24
rs1203952	20	22562132	*FOXA2*	G	GWAS	0.28	0.21	1.49 (1.21-1.83)	1.37×10^{-4}
					Italian	0.27	0.23	1.25 (1.11-1.41)	2.10×10^{-4}
rs4812092	20	57539968	*LOC645605*	T	GWAS	0.23	0.16	1.56 (1.25-1.95)	9.17×10^{-5}
					Italian	0.22	0.22	1.00 (0.88-1.13)	0.97

(*) Deviation from Hardy-Weimberg equilibrium in controls (*p-value*<0.005)

Supplementary Table S3. *In silico* predictions for the high LD SNPs with the strongest associated variants

**dbSNP ID**	**r^2 [(a)]**	**Closest gene annotated**	**Regulatory proteins bound [(b)]**	**Motifs changed (TFs)[(b)]**	**Chromatin structure (cell types)[(b)]**	**Enhancer histone markers (cell types)[(b)]**	**Promoter histone markers (cell types)[(b)]**	**eQTL analysis *p-value*[(c)]**
rs146114442	0.87	*GALNTL4*	-	AP-1; FXR	-	HMEC	-	NA
rs55776195	0.98	*GALNTL4*	-	E2F; EWSR1-FLI1	Osteobl	HSMM	-	NA
rs7934617	1	*GALNTL4*	-	RFX5; STAT	-	HSMM; HMEC	-	0.03
rs7944567	1	*GALNTL4*	-	NF-AT; NF-E2; Pax-4	-	HSMM; HMEC; H1	-	0.06
**rs7935113**	**1**	***GALNTL4***	**-**	**MZF1::1-4; SRF**	**H1-hESC; HMEC; 8988T; iPS; HA-h; HRCEpiC; NT2-D1**	**HSMM; H1**	**-**	**0.03**
rs72867112	0.95	*GALNTL4*	-	NRSF	-	-	-	NA
rs7101505	0.95	*GALNTL4*	-	E2A; Pbx3; Smad	-	-	-	NA
rs72867113	0.92	*GALNTL4*	-	AP-1	-	-	-	NA
rs7102213	0.95	*GALNTL4*	-	BHLHE40; CTCF; ERalpha-a; Pou2f2; ZEB1	iPS	-	-	0.04
rs55860905	0.95	*GALNTL4*	-	-	-	-	-	NA
rs7946349	0.95	*GALNTL4*	-	GCNF; HNF4; RXRA; Smad	-	-	-	NA
rs7946620	0.94	*GALNTL4*	-	GR; Zfp187	-	GM12878	-	NA
rs7928260	0.95	*GALNTL4*	-	AP-4; HEN1	-	GM12878	-	3.40×10^{-3}
rs7946738	0.95	*GALNTL4*	-	Pax-1	-	GM12878	-	9.90×10^{-3}
rs7928402	0.95	*GALNTL4*	-	Ik-2; Mef2; Pou5f1	-	GM12878	-	0.02
rs1203920	0.94	*FOXA2*	-	Irx	-	-	-	NA
rs1203921	0.95	*FOXA2*	-	TBX5; TCF4	-	-	-	3.7×10^{-6}
rs1203922	0.95	*FOXA2*	MAFF; MAFK	RBP-Jkappa; TCF11::MafG	-	-	-	1.6×10^{-6}
rs1203923	0.96	*FOXA2*	-	-	-	-	-	3.7×10^{-6}
rs1203924	0.95	*FOXA2*	-	NRSF	-	-	-	1.6×10^{-6}
rs11087391	0.95	*FOXA2*	-	Hoxa5; Smad4	-	-	-	NA
rs1007458	0.97	*FOXA2*	-	Mef2; Sox	-	-	-	3.7×10^{-6}
rs995734	0.97	*FOXA2*	-	CEBPA; Ik-1; Ik-2; Maf; STAT	H7-hESC	-	-	3.7×10^{-6}
rs1203925	0.96	*FOXA2*	-	-	-	-	-	1.6×10^{-6}
rs1203926	0.94	*FOXA2*	-	Arid3a; CTCF; Dbx1; Foxj2; HDAC2; Irf; Mef2; Ncx; PLZF; TATA; Zfp105	-	-	-	3.4×10^{-6}
rs1007202	0.97	*FOXA2*	-	GZF1; Hic1	-	-	-	3.7×10^{-6}
rs1203927	0.96	*FOXA2*	-	BDP1; GR; NRSF; Pbx3; Spz1; UF1H3BETA; VDR; WT1	-	-	-	NA
rs1203928	0.97	*FOXA2*	-	DMRT7; Znf143	-	-	-	3.6×10^{-6}
rs1203929	0.96	*FOXA2*	-	Gfi1b	-	-	-	1.5×10^{-6}
rs2424435	0.96	*FOXA2*	-	Egr-1; GLI; Klf7; SP1; Sp4	-	-	-	3.1×10^{-6}
rs2424436	0.96	*FOXA2*	-	AhR::Arnt; Arnt; Egr-1; Klf7; Pax-4; SP1; Sp4; ZID; Zfp161	-	-	-	3.1×10^{-6}
rs1203930	0.96	*FOXA2*	-	-	-	-	-	1.5×10^{-6}
rs1203931	0.97	*FOXA2*	-	-	-	-	-	3.6×10^{-6}
rs1203932	0.97	*FOXA2*	-	FAC1	-	-	-	3.6×10^{-6}
rs1203933	0.96	*FOXA2*	-	INSM1; NRSF; RREB-1;Smad3	-	-	-	1.5×10^{-6}
rs1203934	0.97	*FOXA2*	-	-	-	-	-	3.7×10^{-6}
rs1203935	0.96	*FOXA2*	-	FAC1	-	-	-	NA
rs1203936	0.96	*FOXA2*	-	-	H1-hESC; H7-hESC; NT2-D1	-	-	3.6×10^{-6}
rs1203937	0.96	*FOXA2*	-	HNF1; HP1-site-factor; Hoxc10; Nkx2	-	-	-	3.6×10^{-6}
rs1203938	0.96	*FOXA2*	-	CIZ; Foxa; Gfi1; STAT; p300	-	-	-	2.0×10^{-6}
rs1203939	0.96	*FOXA2*	-	Gfi1; Gfi1b; NRSF; Rad21; ZBRK1	-	-	-	2.0×10^{-6}
rs1203940	0.97	*FOXA2*	-	Pax-5	-	-	-	3.6×10^{-6}
rs61186493	0.94	*FOXA2*	-	Barhl1; Barx1; Barx2; Bsx; Dbx1; Dbx2; Esx1; GATA; Hmx;	-	-	-	NA

				Hoxb8; Lhx3; NF-AT; Ncx; Nkx6-1; Nrf-2; Pax-6; Pou4f3; Prrx2; Sox				
rs1203941	0.97	*FOXA2*	-	Elf5; FEV; GR; PU-1; Tel2	HEEpiC	HMEC	-	$3.6{\times}10^{-6}$
rs1203942	0.97	*FOXA2*	-	Crx; Pitx2; Sin3Ak-20	-	HMEC	-	$3.6{\times}10^{-6}$
rs1203943	0.97	*FOXA2*	-	Arid3a; Bbx; Dbx1; HNF1; Hmbox1; Hoxd10; Lhx3; Nkx2; Nkx6-1; Pou3f4; Sox_3	-	HMEC	-	$3.6{\times}10^{-6}$
rs1203944	0.95	*FOXA2*	-	GATA	-	HMEC	-	$2.1{\times}10^{-6}$
rs1203945	0.96	*FOXA2*	-	AP-1	-	HMEC	-	$3.9{\times}10^{-6}$
rs1203946	0.96	*FOXA2*	-	Hbp1; Mef2	iPS	H1	-	NA
rs1203947	0.96	*FOXA2*	-	Gfi1; NF-E2	-	-	-	$1.6{\times}10^{-6}$
rs1203948	0.94	*FOXA2*	-	LUN-1	-	-	-	$1.6{\times}10^{-6}$
rs1203949	0.97	*FOXA2*	-	CEBPB; Cdx; Nanog; Ncx; Nkx2; Pou5f1; Sox	-	-	-	$1.5{\times}10^{-6}$
rs1146365	0.97	*FOXA2*	-	_known4	-	-	-	NA
rs1203950	0.97	*FOXA2*	-	Maf; Pou2f2	-	-	-	NA
**rs1203952**	**1**	***FOXA2***	**FOXA1**	**Evi-1; Foxp1; Pou2f2; SIX5**	**MCF-7**	**-**	**-**	$\mathbf{1.5{\times}10^{-6}}$
rs1203953	0.97	*FOXA2*	-	CHOP::CEBPalpha; DMRT3; DMRT7	-	-	-	$7.6{\times}10^{-7}$
rs6137704	0.92	*FOXA2*	-	Arid5a;Foxa; Mef2; Nkx3; Pou2f2; TATA	-	-	-	$7.8{\times}10^{-7}$
rs2424439	0.96	*FOXA2*	-	-	-	-	-	$7.7{\times}10^{-7}$
rs2208168	0.93	*FOXA2*	-	TFIIA	-	-	-	$9.0{\times}10^{-7}$
rs2424440	0.93	*FOXA2*	-	Arid5b; Evi-1; Pou2f2	-	-	-	$8.2{\times}10^{-6}$
rs2208169	0.93	*FOXA2*	-	NF-I; VDR	-	-	-	NA
rs2424442	0.93	*FOXA2*	-	Crx; Dmbx1; Gsc; Rad21	-	-	-	$9.0{\times}10^{-7}$
rs6082762	0.94	*FOXA2*	SETDB1	RXRA	-	-	-	$9.0{\times}10^{-7}$
rs2424443	0.93	*FOXA2*	SETDB1	PLAG1	-	-	-	$9.0{\times}10^{-7}$
rs6075931	0.93	*FOXA2*	-	PU-1; TCF12; Zfx	H7-hESC	-	-	$9.0{\times}10^{-7}$
rs910960	0.88	*FOXA2*	-	CEBPA; CEBPB; DMRT2; Evi-1; HDAC2; Irf; RFX5; STAT	-	-	-	NA
rs1884742	0.9	*FOXA2*	-	LUN-1	-	-	-	$3.0{\times}10^{-6}$
rs6137712	0.89	*FOXA2*	-	Pou5f1	-	-	-	NA
rs2424444	0.9	*FOXA2*	-	Nanog; Pou5f1	-	-	-	NA
rs8118625	0.9	*FOXA2*	-	Evi-1; HDAC2; HNF1; Hoxa10; Hoxa9; Hoxb13; Hoxd10; PLZF; TATA; Zfp105	-	-	-	NA
rs2224316	0.9	*FOXA2*	-	Pax-4	-	-	-	NA
rs6082765	0.9	*FOXA2*	-	Foxj1; Pbx-1	-	-	-	NA
rs2180553	0.91	*FOXA2*	-	NRSF; Sin3Ak-20	-	-	-	$4.4{\times}10^{-7}$
rs747911	0.9	*FOXA2*	-	GATA; TATA	-	-	-	NA
rs747912	0.91	*FOXA2*	-	-	-	-	-	$9.0{\times}10^{-7}$
rs910956	0.9	*FOXA2*	-	Pax-5; SP1	-	-	-	NA
rs754855	0.88	*FOXA2*	-	BCL; CTCFL; EBF; INSM1; NF-I; NRSF; PU-1; Sin3Ak-20; TCF12	HMEC	HSMM; H1	-	NA
rs910957	0.85	*FOXA2*	-	-	A549; HeLa-S3; FibroP	H1	HSMM	NA
rs4815129	0.83	*FOXA2*	-	AP-1	-	-	-	NA

[a]) LD value between each SNP and the SNP of interest (in bold)

[b] Functional annotations from the ENCODE-based tool HaploReg v2 (www.broadinstitute.org/mammals/haploreg)

[c] eQTL analysis on lymphoblastoid cells according to SNPexp (http://tinyurl.com/snpexp) for the LD block including rs7935113 and on thyroid tissues according to GTEx Portal (http://www.gtexportal.org/home/) for the LD block including rs1203952.

Supplementary Table S4. Information on the SNPs analyzed for their cumulative risk.

SNP	Chr	Position	Gene	Risk allele	Cohort	Allelic OR (95% CI)	*p-value*
rs6759952	2	217979964	*DIRC3*	T	GWAS	1.44 (1.21-1.72)	4.7×10^{-5}
					Italian1	1.32 (1.17-1.49)	9.8×10^{-6}
					Italian2	1.16 (0.96-1.41)	0.12
rs10238549(*)	7	109968258	*IMMP2L*	C	GWAS	1.48 (1.23-1.77)	3.2×10^{-5}
					Italian1	1.21 (1.06-1.37)	4.7×10^{-3}
					Italian2	1.16 (0.94-1.42)	0.16
rs7800391(*)	7	109995479	*IMMP2L*	T	GWAS	1.45 (1.21-1.73)	5.2×10^{-5}
					Italian1	1.22 (1.07-1.38)	2.3×10^{-3}
					Italian2	1.06 (0.87-1.28)	0.55
rs7617304(*)	3	159945795	*RARRES1*	A	GWAS	1.37 (1.13-1.67)	1.4×10^{-3}
					Italian1	1.16 (1.01-1.34)	0.03
					Italian2	1.22 (1.00-1.50)	0.05
rs10781500(*)	9	138389159	*SNAPC4/CARD9*	C	GWAS	1.51 (1.23-1.86)	7.9×10^{-5}
					Italian1	1.18 (1.04-1.34)	0.01
					Italian2	1.20 (0.98-1.46)	0.07
rs10136427	14	75049642	*BATF*	C	GWAS	1.62 (1.28-2.06)	5.73×10^{-5}
					Italian	1.26 (1.09-1.45)	1.32×10^{-3}
rs7267944	20	37380848	*DHX35*	C	GWAS	1.54 (1.24-1.90)	6.60×10^{-5}
					Italian	1.29 (1.14-1.45)	5.83×10^{-5}
rs13184587(*)	5	78242539	*ARSB*	G	GWAS	1.51 (1.24-1.83)	3.60×10^{-5}
					Italian	1.19 (1.06-1.33)	4.15×10^{-3}
rs1220597(*)	13	23716013	*SPATA13*	C	GWAS	1.42 (1.20-1.70)	7.11×10^{-5}
					Italian	1.20 (1.08-1.33)	5.02×10^{-4}
rs7935113(*)	11	11492456	*GALNTL4*	C	GWAS	1.50 (1.20-1.88)	3.26×10^{-4}
					Italian	1.28 (1.12-1.46)	2.20×10^{-4}
rs1203952(*)	20	22562132	*FOXA2*	G	GWAS	1.49 (1.21-1.83)	1.37×10^{-4}
					Italian	1.25 (1.11-1.41)	2.10×10^{-4}

(*) SNPs associated with DTC risk only in the Italian cohorts

Supplementary Table S5. Risk of DTC associated with increasing number of risk alleles of eleven SNPs; combined analysis of the GWAS and the Italian replication study

Number of risk alleles	Cases	Controls	OR (95% CI)
≤ 7	103 (5.75%)	243 (15.30%)	Reference
8	153 (8.54%)	221 (13.92%)	1.63 (1.20-2.23)
9	249 (13.90%)	303 (19.08%)	1.94 (1.46-2.58)
10	308 (17.20%)	294 (18.51%)	2.47 (1.87-3.27)
11	322 (17.98%)	223 (14.04%)	3.41 (2.56-4.54)
12	271 (15.13%)	157 (9.89%)	4.07 (3.01-5.51)
13	193 (10.78%)	88 (5.54%)	5.17 (3.68-7.28)
≥ 14	192 (10.72%)	59 (3.72%)	7.68 (5.29-11.13)
Total	1,791 (100%)	1,588 (100%)	1.30 (1.26-1.35) per allele *p-trend*=3.13×10^{-47}

Supplementary Table S6. Risk of DTC associated with increasing number of risk alleles of twelve SNPs (including *FOXE1* rs965513); the analysis is based on the GWAS samples only

Number of risk alleles	Cases	Controls	OR (95%CI)
≤ 7	14 (2.18%)	49 (11.78%)	Reference
8	28 (4.36%)	59 (14.18%)	1.66 (0.79-3.50)
9	54 (8.41%)	65 (15.63%)	2.91 (1.45-5.83)
10	78 (12.15%)	76 (18.27%)	3.59 (1.83-7.04)
11	111 (17.29%)	68 (16.35%)	5.71 (2.93-11.12)
12	104 (16.20%)	48 (11.54%)	7.58 (3.82-15.05)
13	104 (16.20%)	32 (7.69%)	11.38 (5.57-23.23)
≥ 14	149 (23.21%)	19 (4.57%)	27.45 (12.81-58.81)
Total	642 (100%)	416 (100%)	1.52 (1.42-1.63) per allele *p-trend*=1.71×10^{-32}

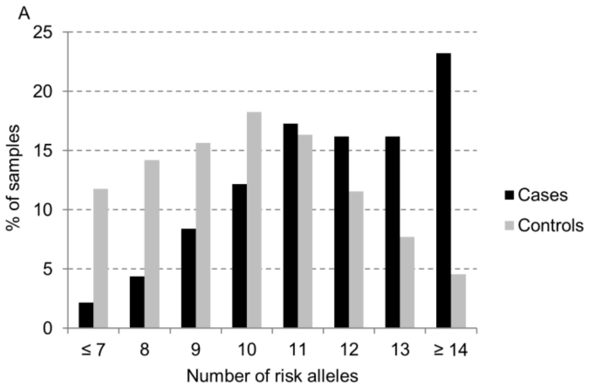

Supplementary Figure S1. Cumulative risk assessment including the FOXE1 SNP rs965513. (A) Sample distribution according to the number of risk alleles in twelve SNPs associated in the Italian DTC cases (black columns) and controls (grey columns). (B) Plot of the increasing ORs for DTC with increasing number of risk alleles. The category ≤7 was chosen as reference; vertical bars correspond to 95% confidence intervals. Horizontal line denotes the null value (OR=1.0).
